# pH-responsive self-assembly of quercetin-loaded zein-sodium caseinate-fucoidan hybrid nanoparticles: nanostructure, stability and *in vitro* digestive behavior

**DOI:** 10.3389/fnut.2026.1727771

**Published:** 2026-01-28

**Authors:** Fan-Xing Yong, Jia-Xin Deng, Zhuo Wang, Qiao-Li Zhao, Xiao-Fei Liu, Sai-Yi Zhong, Rui Li

**Affiliations:** 1 Guangdong Provincial Key Laboratory of Aquatic Product Processing and Safety, Guangdong Province Engineering Laboratory for Marine Biological Products, Guangdong Provincial Engineering Technology Research Center of Seafood, Guangdong Provincial Engineering Technology Research Center of Prefabricated Seafood Processing and Quality Control, Guangdong Provincial Science and Technology Innovation Center for Subtropical Fruit and Vegetable Processing, College of Food Science and Technology, Guangdong Ocean University, Zhanjiang, China; 2 College of Food Science and Technology, Huazhong Agricultural University, Wuhan, China; 3 Shenzhen Research Institute, Guangdong Ocean University, Shenzhen, China

**Keywords:** fucoidan, nanoparticle, pH-driven, quercetin, sodium caseinate, zein

## Abstract

**Introduction:**

Quercetin (Que), a physiologically versatile flavonoid, faces application limitations in food and pharmaceuticals due to poor aqueous solubility and stability.

**Method:**

To address this, we developed quercetin-loaded zein-sodium caseinate-fucoidan (Que-ZE-SC-FD) ternary nanoparticles using a green, pH-driven approach.

**Results and discussion:**

The Que-ZE-SC-FD nanoparticle exhibited a spherical morphology stabilized by hydrogen bonding, electrostatic, and hydrophobic interactions, with a mean diameter of 137.8 ± 11.6 nm, PDI of 0.38 ± 0.04, z-potential of 34.9 ± 0.6mV, and high quercetin loading efficiency (92.8% ± 1.1%). Crucially, SC and FD demonstrated synergistic stabilization effects. The Que-ZE-SC-FD nanoparticle exhibited a mean particle size of 150.8 ± 0.6 nm at a pH of 8.0, and solution remained clear and transparent with no observable sediment. Under a NaCl concentration of 3.0 mol/L, the particle size decreased to 127.8 ± 4.5 nm. Upon heating at 80°C for 2 h, the particle size further reduced to 121.3 ± 1.2 nm, with a PDI of 0.34 ± 0.02. After 28 days of storage, the particle size decreased to 125.1 ± 1.9 nm, while the PDI decreased slightly to 0.32 ± 0.01 and the zeta potential increased to 31.6 ± 1.5mV, collectively indicating excellent stability. Under simulated gastrointestinal conditions, the Que release from ZE-SC-FD nanoparticles was only 22.2 ± 0.5% in gastric fluid; however, a significantly higher release rate of 75.0 ± 0.5% was achieved in intestinal fluid. These results demonstrate that ZE-SC-FD nanoparticles serve as a robust nanocarrier system for encapsulating, protecting, and delivering quercetin.

## Graphical Abstract

**Figure:**
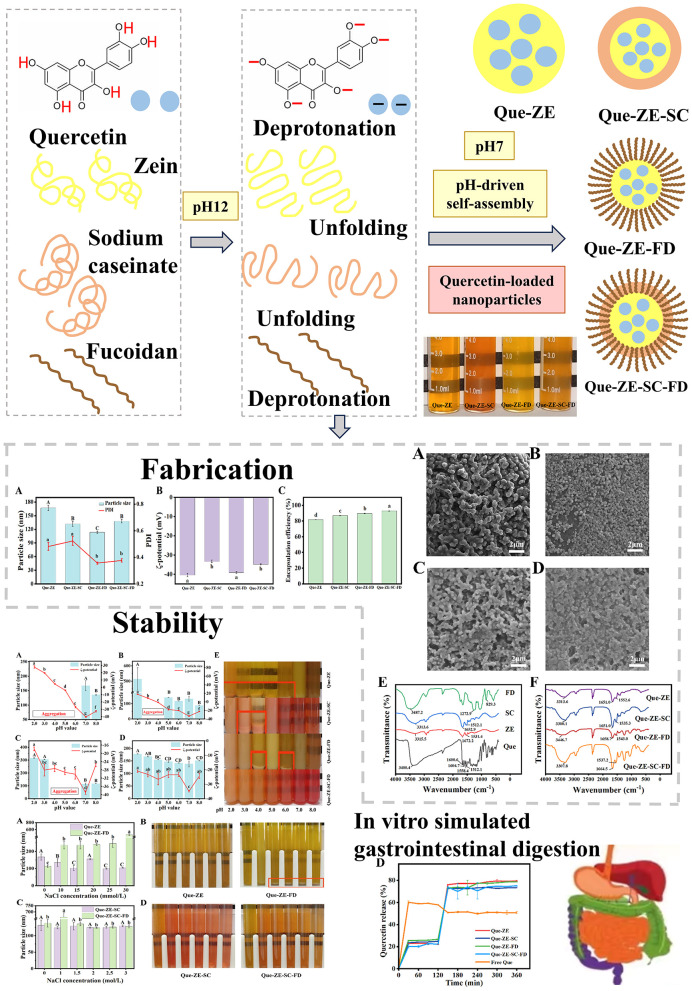

## 1 Introduction

Quercetin [3,5,7-trihydroxy-2-(3,4-dihydroxyphenyl)-4H-chromen-4-one, Que], a ubiquitous flavonoid aglycone, is abundant in vegetables, fruits, and grains such as sorghum, oats, and wheat (1). As a representative flavonoid antioxidant (2), quercetin exhibits diverse biological activities, including anti-inflammatory (3), anti-diabetic (4), and neuroprotective effects (5), along with potential for mitigating infections caused by SARS-CoV-2 variants (6). Despite its promising bioactivity, the practical application of quercetin in functional foods and pharmaceuticals is constrained by inherent physicochemical challenges, notably low aqueous solubility, poor chemical stability, limited gastrointestinal absorption (7).

To overcome these limitations, various nanocarriers have been explored for quercetin encapsulation, including nano-liposomes (8), nano-emulsions (9), hydrogels (10), cyclodextrin inclusion complexes (11), and nanoparticles (12). Notably, nanoparticles offer distinct advantages for enhancing the delivery of bioactive compounds like quercetin. Specifically, they enhance aqueous solubility and dissolution kinetics (13), protect encapsulated cargo from degradation in harsh biological environments (e.g., gastric fluids, light, and oxygen) (14), improve cellular uptake and permeability across biological barriers (15), and enable controlled or targeted release profiles (12).

Zein (ZE), an alcohol-soluble plant protein derived from corn, is regarded as a highly promising nanoparticle material. It is non-toxic, biocompatible, and has been granted Generally Recognized As Safe (GRAS) status by the U.S. Food and Drug Administration (16). Comprising over 50% hydrophobic amino acids (e.g., proline, leucine, and alanine), zein possesses a hydrophilic structure with distinct hydrophobic and hydrophilic domains (17). This amphiphilicity underpins its insolubility in water but solubility in aqueous ethanol (18), and enables the self-assembly of nanoparticles capable of encapsulating hydrophobic actives like quercetin via hydrogen bonding, hydrophobic interactions, and electrostatic forces, thereby enhancing their stability and bioavailability (19). While various strategies have been proposed to enhance the water solubility and stability of quercetin, but usually organic solvents are required, such as dimethyl sulfoxide (precipitation techniques) (20) and ethanol (anti-solvent precipitation method) (21), which raises cost and safety concerns, also might limit its use in food industry. Therefore, a low-energy and organic solvent-free strategy was urgently needed to improve the dispersion and stability of quercetin. The pH-driven method presents as an efficient, environmentally friendly alternative (22). This technique involves dissolving zein in a highly alkaline solution, followed by adjustment to neutrality or acidity. As the pH decreases, zein undergoes conformational changes, leading to decreased solubility and spontaneous self-assembly into nanoparticles with a hydrophobic core (23). This solvent-free approach is advantageous due to its simplicity, low cost, low energy consumption, and environmental benignity, and has proven effective for the delivery of polyphenol (24, 25).

However, zein nanoparticles alone exhibit limited stability. They are susceptible to aggregation, denaturation, or degradation under challenging conditions, such as pH near the protein's isoelectric point (pI), elevated ionic strength, or exposure to proteolytic enzymes (26). Their inherent hydrophobicity also contributes to poor resistance against aggregation (27). Enhancing the stability of zein nanoparticles is therefore a critical research focus (28). In this context, surface coating has been established as an effective stabilization strategy (29). For instance, sodium caseinate (SC), a water-soluble milk protein, can be applied as a coating material via electrostatic deposition, where it functions effectively as a natural stabilizer for zein nanoparticles (30, 31). Nevertheless, Zein-SC complexes still face stability challenges, particularly at pH values approaching the protein's pI, indicating a need for further improvement (32, 33).

Natural polysaccharides have also shown promise as auxiliary stabilizers for zein nanoparticles by enhancing electrostatic and steric repulsion (34). Examples include carrageenan (32), chitosan (35), and alginate (36), which have been shown to significantly enhance nanoparticle stability through surface coating. Fucoidan (FD), a naturally occurring sulfated anionic polysaccharide, exhibits excellent water solubility and facilitates nanoparticle formation through its ionizable sulfate groups in acidic solutions (26). Fucoidan readily forms stable nanocomplexes with various proteins [e.g., soy protein isolate (37), fish protamine (38)] via electrostatic interactions. Furthermore, fucoidan possesses superior solubility, low viscosity, and high charge density across a wide pH range (39). However, to the best of our knowledge, the development and characterization of ternary nanocomposites comprising zein, sodium caseinate, and fucoidan for quercetin encapsulation have remained largely unexplored.

Therefore, this study aims to develop quercetin-loaded nanoparticles based on the pH-induced denaturation and renaturation mechanism of zein, employing a simple, green and low-cost pH-driven co-assembly strategy. Building upon previous research, a ZE:SC:FD mass ratio of 1:1:1 was selected as the optimal formulation (40). Under this ratio, the nanoparticles exhibit a narrow particle size distribution, a low PDI value, and a relatively high surface charge. We systematically investigated the impact of SC and fucoidan incorporation on the physical properties of zein-based nanoparticles, including particle size, ζ-potential, microstructure, and quercetin encapsulation efficiency (EE). The potential formation mechanism of the complex and the interactions between its components were explored. Furthermore, the physical stability of the nanoparticles was rigorously evaluated under diverse stress conditions (pH, ionic strength, and storage time). Finally, the *in vitro* release profiles of quercetin during simulated gastrointestinal digestion were assessed. This work not only advances the green fabrication of zein-based nanoparticles but also provides a theoretical foundation for expanding the applications of the ZE-SC-FD ternary complex in functional foods and nutraceutical delivery.

## 2 Materials and methods

### 2.1 Materials

Quercetin (purity 99%) was purchased from Aladdin Holding Group Co., Ltd. (Shanghai, China). Zein, sodium caseinate, and fucoidan were obtained from Sigma Aldrich Trading Co., Ltd. (Shanghai, China), Shanghai Maclin Biochemical Technology Co., Ltd. (Shanghai, China), and Qingdao Bright Moon Hailin Fucoidan Bio-Tech Co., Ltd. (Qingdao, China). Pepsin, bile salt and trypsin were sourced from Beijing Solarbio Science & Technology Co., Ltd. (Beijing, Chian). All organic solvents used for separation were analytical grade.

### 2.2 Preparation of composite nanoparticles

#### 2.2.1 Preparation of stock solution

Zein-based nanoparticles were prepared using a pH-driven method. First, a zein stock solution (10 mg/mL) was prepared by dissolving 1.0 g of zein in 100 mL of 10 mmol/L NaOH aqueous solution. The same to sodium caseinate and fucoidan stock solutions (each 10 mg/mL). All stock solutions were freshly prepared immediately before nanoparticle fabrication.

#### 2.2.2 Preparation of four nanoparticles

Quercetin-loaded nanoparticles were prepared via pH-driven self-assembly method, following an optimized formulation reported previously (40), with ZE, SC, and FD incorporated at a 1:1:1 mass ratio. Briefly, 50 mg of quercetin was dissolved in the zein stock solution under dark conditions with continuous stirring (500 r/min, 12 h, 25°C) to ensure complete solubilization. The quercetin-loaded zein solution was then separately mixed with different combinations of polysaccharides: (Que-ZE) 20 mL NaOH (Que-ZE-SC) 10 mL SC + 10 mL NaOH (Que-ZE-FD) 10 mL FD + 10 mL NaOH, and (Que-ZE-SC-FD) 10 mL SC + 10 mL FD, followed by the addition of 30 mL citric acid solution (3 mmol/L) to adjust the pH to 7.0 ± 0.1, thereby inducing nanoparticle formation. The resulting colloidal dispersions were centrifuged (5000 r/min, 10 min, 25°C) to eliminate aggregates and unbound components. The supernatants were collected as purified nanoparticle suspensions designated as Que-ZE, Que-ZE-SC, Que-ZE-FD, and Que-ZE-SC-FD, respectively. The final concentrations of ZE, SC, and FD were all 16.7 mg/mL, respectively.

### 2.3 Characterization of composite nanoparticles

#### 2.3.1 Particle size and ζ-potential

The characteristic parameters of the nanoparticles, including average particle size, polydispersity index (PDI), and ζ-potential were measured using a nanoparticle size and Zeta potentiometer (Zetasizer Nano ZSE, Malvern, UK).

#### 2.3.2 Encapsulation efficiency (EE)

The encapsulation efficiency was determined according to the method described by Wei et al. (41) with slight modifications. Four types of prepared composite nanoparticles (1 mL) were mixed with anhydrous ethanol (2 mL). The mixture was vortexed at 1500 r/min for 2 min and then centrifuged at 7500 r/min for 10 min. Absorbance was measured at 370 nm using a UV–vis spectrophotometer. The concentration was calculated based on the Que standard curve, which was established under the same experimental conditions: y = 15.53x + 0.000776 (*R*^2^ = 0.9999).

The EE of Que was calculated using Equation (1):


$$\begin{array}{l} EE\left(\%\right)=\frac{m_{0}}{m_{1}}\times 100\% \end{array}$$ (1)

Here, m_0_ was the mass of quercetin in the nanoparticles, and m_1_ denoted the total mass of quercetin added to the stock solution before encapsulation.

#### 2.3.3 Scanning electron microscopy (SEM)

Nanoparticle morphology was characterized using scanning electron microscopy (SEM; TESCAN MIRA3, Brno, Czech Republic). Freeze-dried powder samples were mounted on stainless steel stubs using conductive adhesive and sputter-coated with gold under vacuum (5 kV acceleration voltage, 60 s coating time). Microstructural analysis was performed at an accelerating voltage of 5 kV under high vacuum.

#### 2.3.4 Fourier transform infrared spectroscopy (FT-IR)

Functional groups were analyzed using FT-IR spectroscopy (BRUKER TENSOR II, Ettlingen, Germany) following a modified protocol from Li et al. (42). Freeze-dried nanoparticles (1~2 mg) were homogenously blended with dried KBr powder (1:100, w/w) in an agate mortar until particle size reached ≤ 2 μm. The mixture was compressed at 15 MPa for 1–2 min to form transparent pellets. Spectra were acquired at 4 cm^−1^ resolution with 32 scans the 4000~400 cm^−1^ range, with background subtraction performed using pure KBr pellets.

### 2.4 Stability evaluation

#### 2.4.1 pH and ionic strength stability

pH stability was assessed by adjusting Que-loaded nanoparticle dispersions to pH 2.0–8.0 using 100 mmol/L HCl or NaOH. Ionic strength stability was evaluated by mixing equal volumes of Que-ZE and Que-ZE-FD dispersions with NaCl solutions (final concentrations: 0–30 mmol/L); Que-ZE-SC and Que-ZE-SC-FD nanoparticles were mixed with NaCl solutions (final concentrations 0–3.0 mol/L) (40), based on caseinate's established anti-aggregation properties (43). All samples were equilibrated at 25°C for 24 h before measuring hydrodynamic diameter and ζ-potential by dynamic light scattering (Zetasizer Nano ZSE, Malvern Instruments, UK).

#### 2.4.2 Thermal stability

To assess the thermal stability of quercetin-loaded nanoparticles, freshly prepared dispersions (30 mL) of Que-ZE, Que-ZE-SC, Que-ZE-FD, and Que-ZE-SC-FD nanoparticles were subjected to heating in an 80°C water bath for varying durations (0~120 min). Following heat treatment, samples were cooled to room temperature (25°C) and equilibrated for 24 h prior to particle size and ζ-potential measurements.

#### 2.4.3 Storage stability

Freshly prepared Que-ZE, Que-ZE-SC, Que-ZE-FD, and Que-ZE-SC-FD nanoparticle dispersions were stored in darkness at 4°C. The particle size and ζ-potential were measured in triplicate at 0, 7, 14, and 28 d.

### 2.5 *In vitro* simulated gastrointestinal digestion

Release profiles of free quercetin and quercetin-loaded nanoparticles were evaluated using a modified protocol (44, 45). The procedure comprised gastric phase: 30 mL nanoparticle dispersion was mixed with 30 mL simulated gastric fluid [simulated gastric fluid (SGF): pepsin 3.2 mg/mL, NaCl 2 mg/mL, pH 2.0]. After incubation (37°C, 100 r/min, 2 h), 2 mL aliquots were sampled at 30-min intervals with SGF replenishment. At intestinal phase, the gastric digest (60 mL) was combined with 60 mL simulated intestinal fluid (SIF: trypsin 2.4 mg/mL, porcine bile salt 5 mg/mL, NaCl 120 mmol/L, CaCl_2_ 10 mmol/L, pH 7.0). Following incubation (37°C, 400 r/min, 4 h), 2 mL samples were collected hourly with SIF replacement. All samples were centrifuged (10,000 r/min, 10 min). Supernatant were diluted 1:1 (v/v) with 80% ethanol and analyzed at 373 nm. Cumulative release was calculated utilizing Equation (2):


$$\begin{array}{l} \text{Release rate}\left(\text{\%}\right)=\frac{C}{C_{0}}\times 100\% \end{array}$$ (2)

Here, *c* was the amount of quercetin in the digestion solution, and c_0_ was the amount of initial quercetin concentration in the nanodispersion or free quercetin before digestion.

### 2.6 Statistical analysis

All the experiments were performed three times, and the results are expressed as mean ± standard deviations (SD). Significant differences (*p* < 0.05) were determined by one-way ANOVA with Duncan's *post-hoc* test using SPSS (v27.0, IBM Corp.).

## 3 Results and discussion

### 3.1 Characterizations of quercetin-loaded composite nanoparticles

#### 3.1.1 Particle size, PDI, ζ-potential and EE

**Figure 1:**
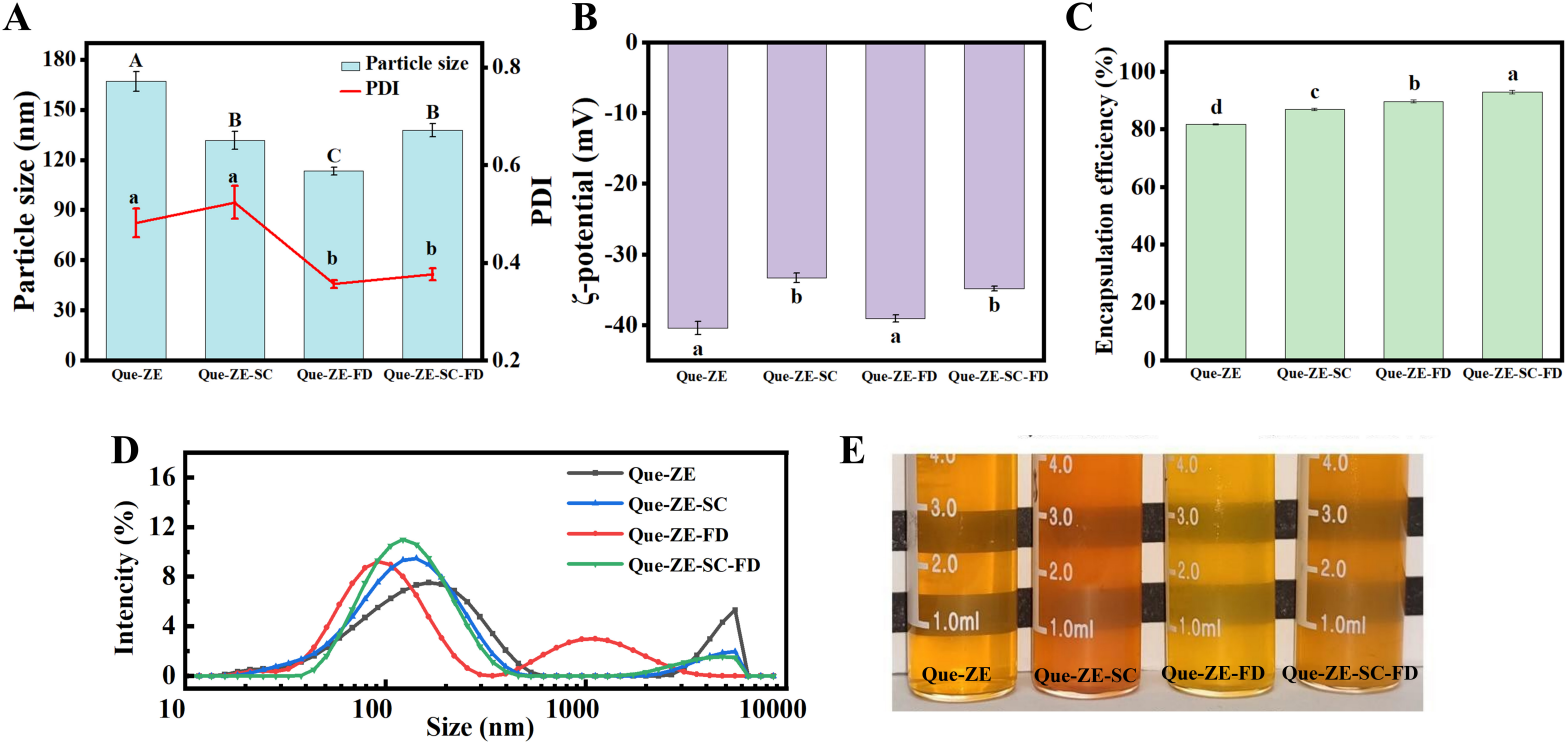
Mean particle size and PDI **(A)**, ζ-potential **(B)**, encapsulation efficiency **(C)**, particle size distribution **(D)** and visual appearance **(E)** of Que-ZE, Que-ZE-SC, Que-ZE-FD, and Que-ZE-SC-FD nanoparticles.

In this study, Que-ZE Serves as the baseline control, demonstrating the properties of zein nanoparticles alone; Que-ZE-SC and Que-ZE-FD are critical experimental controls that individually demonstrate the stabilizing and encapsulating effects of SC and FD, respectively; The performance of the ternary complex Que-ZE-SC-FD can be directly compared against the binary systems to evaluate synergy. The particle size, polydispersion index (PDI), ζ-potential, and quercetin encapsulation efficiency of Que-ZE, Que-ZE-SC, Que-ZE-FD, and Que-ZE-SC-FD composite nanoparticles are summarized in Figures 1A–C. The mean particle size of Que-ZE was 167.0 ± 17.6 nm with a PDI of 0.48 ± 0.09. Upon incorporation of SC and FD, the particle sizes of Que-ZE-SC, Que-ZE-FD, and Que-ZE-SC-FD decreased significantly (*p* < 0.05) to 131.7 ± 15.9, 113.3 ± 7.3, and 137.8 ± 11.6 nm, respectively. This reduction is likely attributable to the increased hydrophobicity and decreased water solubility of zein induced by SC and FD. Low water solubility promotes a higher degree of supersaturation during nanoparticle formation, thereby enhancing the nucleation rate and favoring smaller particle sizes (46). These observations consistent with the findings in similar systems (47). The PDI of Que-ZE-SC-FD (0.38 ± 0.04) was significantly lower (*p* < 0.05) than that of Que-ZE (0.48 ± 0.09), indicating enhanced suspension uniformity and stability for Que-ZE-SC-FD (48). The ζ-potential values were −40.4 ± 1.6 mV (Que-ZE), −33.4 ± 1.2 mV (Que-ZE-SC), −39.1 ± 0.8 mV (Que-ZE-FD), and −34.9 ± 0.6 mV (Que-ZE-SC-FD). The absolute values of all formulations exceeded 25 mV, suggesting sufficient electrostatic repulsion to minimize nanoparticle aggregation (49). Figure 1C demonstrated that the encapsulation efficiency of Que-ZE is 81.7 ± 0.3%, whereas the encapsulation efficiencies of Que-ZE-SC, Que-ZE-FD, and Que-ZE-SC-FD are increased to 86.8 ± 0.7%, 89.6 ± 0.8%, and 92.8 ± 1.1%, respectively. These results suggest that both sodium caseinate and fucoidan polysaccharide can substantially enhance the encapsulation efficiency of quercetin within zein-based nanoparticles. It should be noted that the measured EE may be higher than the actual value, as it could include surface-adsorbed quercetin.

Consistent trends were observed in the particle size distributions of quercetin-loaded nanoparticles. The peak size distribution for Que-ZE nanoparticles occurred at a larger diameter compared to Que-ZE-SC, Que-ZE-FD, and Que-ZE-SC-FD (Figure 1D). All four nanoparticle suspensions appeared transparent and homogeneous without visible precipitation or aggregation (Figure 1E).

#### 3.1.2 Microscopic morphology analysis

**Figure 2:**
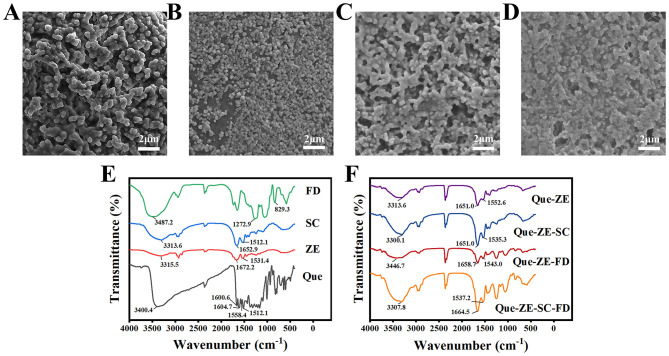
SEM image of Que-ZE **(A)**, Que-ZE-SC **(B)**, Que-ZE-FD **(C)**, and Que-ZE-SC-FD **(D)** nanoparticles and FTIR spectra of pure ingredients (fucoidan, sodium caseinate, zein, and quercetin) **(E)** and quercetin nanoparticles (Que-ZE, Que-ZE-SC, Que-ZE-FD, and Que-ZE-SC-FD nanoparticles) **(F)**.

The morphologies of freeze-dried Que-ZE, Que-ZE-SC, Que-ZE-FD, and Que-ZE-SC-FD nanoparticles were analyzed by scanning electron microscope (SEM; Figures 2A–D, respectively). All samples exhibited spherical or ellipsoidal shapes. Consistent with the particle size data in Figure 1A, Que-ZE particles were significantly larger than those of Que-ZE-SC, Que-ZE-FD, and Que-ZE-SC-FD. However, a distinct tendency for interparticle adhesion was observed. Larger particles appeared to form aggregates resembling grape-like clusters or chains, likely resulting from the coalescence of smaller nanoparticles. Similar aggregation phenomena have been reported previously (50). This behavior may be attributed to quercetin not only encapsulated within the nanoparticles but also partially adsorbed onto their surfaces. The resulting strong hydrophobic interactions between the zein-based nanoparticles are proposed to promote aggregation (51).

#### 3.1.3 FTIR

FTIR analysis (Figures 2E, F) revealed broad absorption peaks within 3100~3500 cm^−1^ for all samples, corresponding to –OH stretching vibrations (52). The –OH peaks for FD, SC, ZE, and Que occurred at 3487.2, 3313.6, 3315.5, and 3400.4 cm^−1^, respectively. Upon formation of the Que-ZE, Que-ZE-SC, Que-ZE-FD, and Que-ZE-SC-FD complexes, these peaks shifted to 3313.6, 3300.1, 3446.7, and 3307.8 cm^−1^, respectively, suggesting the formation of hydrogen bonds during nanoparticle assembly (53). Dai et al. (54) demonstrated that the shift in O-H stretching vibrations in composite nanoparticles indicated hydrogen bonds formed among zein, rhamnolipid, and curcumin. Absorption peaks within 1650~1700 and 1500~1550 cm^−1^ correspond to the amide I and amide II bands, respectively (55). Both ZE and SC exhibited these characteristic amide bands. Significant changes occurred in the amide II region after nanoparticle formation. Compared to ZE (1531.4 cm^−1^) and SC (1512.7 cm^−1^), the amide II band of Que-loaded nanoparticles (Que-ZE, Que-ZE-SC, Que-ZE-FD, and Que-ZE-SC-FD) shifted to 1552.6, 1535.3, 1543.0, and 1537.2 cm^−1^, respectively, indicating electrostatic interactions between ZE, SC, FD, and Que. Li et al. (56) demonstrated that the shifts in the amide I and amide II bands of nanoparticles imply electrostatic interactions formed among luteolin, gum arabic, zein, and tea polyphenols. The FTIR spectrum of free quercetin displayed characteristic benzene ring absorption bands at 1600.6, 1558.4, and 1512.1 cm^−1^ within 750~1600 cm^−1^ (41). Following encapsulation within zein-based nanoparticles, these Que-specific peaks disappeared or were overlapped by nanoparticle absorption bands. Absence of new peaks, precluding covalent bond formation and confirming assembly driven primarily by hydrophobic interactions (57). hydrophobic interactions These findings align with previous reports (58). Compared with Que-ZE, the –OH stretching vibration of Que-ZE-SC-FD exhibits a redshift, and the peak shape becomes broader. The peak in the amide I region undergoes a redshift to 1664.5 cm^−1^, while the peak in the amide II region shows a blueshift to 1537.2 cm^−1^, and the peak shape becomes sharper. The results indicated that the hydrogen bond, electrostatic forces and hydrophobic effects engaged in the interactions of nanoparticles (54, 59).

### 3.2 Stability evaluation

#### 3.2.1 pH stability

**Figure 3:**
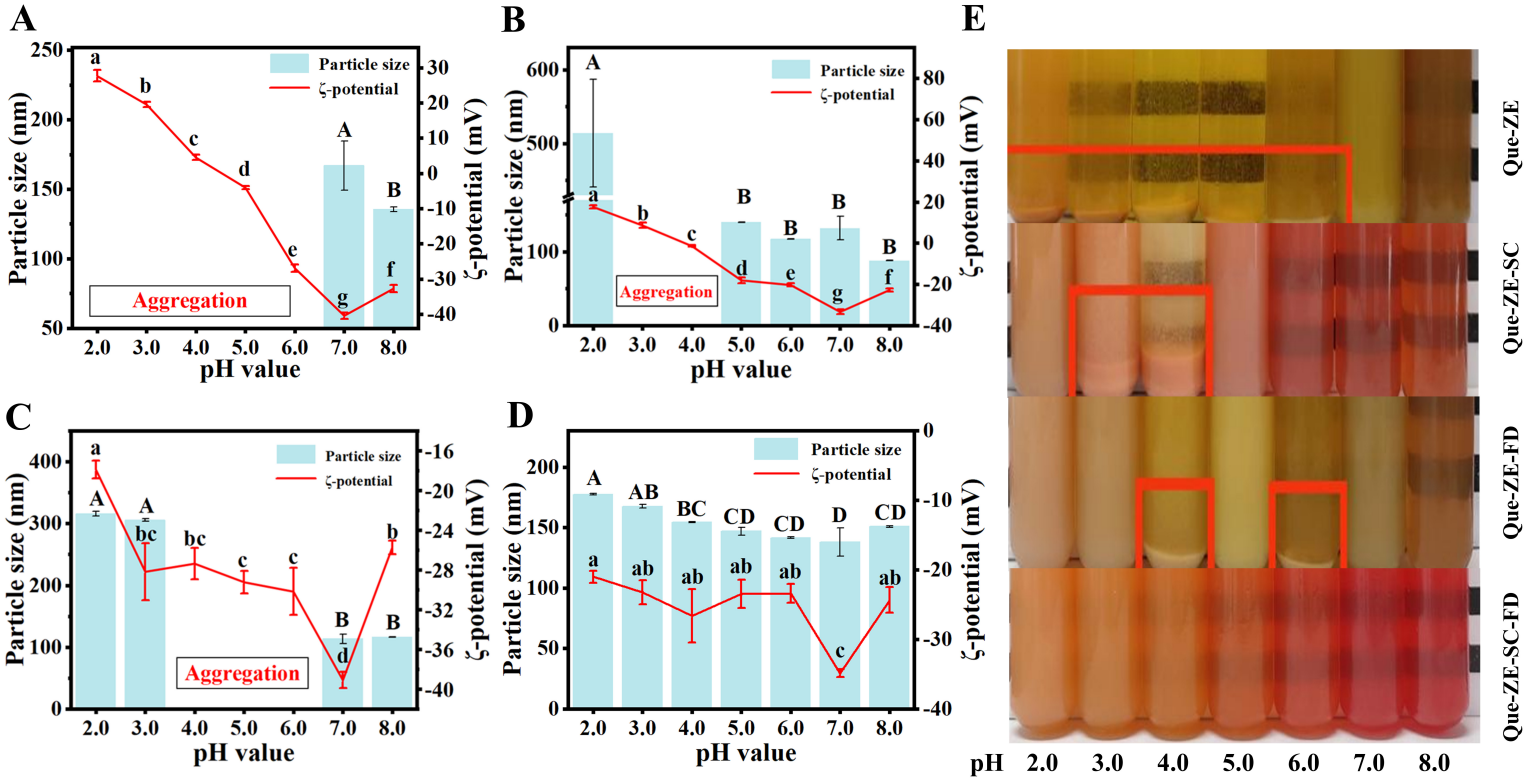
Effects of pH on the mean particle size and ζ-potential of Que-ZE nanoparticles **(A)**, Que-ZE-SC nanoparticles **(B)**, Que-ZE-FD nanoparticles **(C)**, and Que-ZE-SC-FD nanoparticles **(D)**. The photograph illustrates the visual appearance of the samples of each group across the pH range of 2.0 to 8.0 **(E)**. Different letters above the error bars indicate statistically significant differences (*p* < 0.05).

The stability of delivery carriers is critically influenced by pH variations during food production, storage, and consumption, evaluating pH stability is essential. The Que-ZE nanoparticles displayed an isoelectric point (pI) between pH 5.0–6.0 (Figure 3A), consistent with native zein (pH 6.2) (60). Below pH 7.0, these dispersions became unstable, aggregating and precipitating (Figure 3E) due to insufficient electrostatic to overcome hydrophobic attraction (61). The Que-ZE-SC nanoparticles exhibited aggregation within the pH range of 3.0–4.0 (Figure 3B). This instability near the theoretical pI of SC (pH 4.6) (62) suggests neutralization of surface charges and confirms the dominant role of caseinate in forming the surface coating. The dispersion exhibited positive charge at pH 2.0–3.0 and negative charges at pH 4.0–8.0 (Figure 3C), consistent with charge-mediated stabilization mechanisms (63). The Que-ZE-FD nanoparticles showed significant size increase at pH 2.0 (316.0 ± 3.6 nm) and pH 3.0 (305.8 ± 2.3 nm), accompanied by increased turbidity (Figure 3E). This aggregation behavior can be attributed to the proximity to the pKa of the sulfonic acid groups in fucoidan, which reduces electrostatic repulsion (64). In the presence of FD, the ζ-potential of Que-ZE-FD showed a substantially negative charge at pH 2.0–8.0, demonstrating that FU was covered on the Que-ZE-FD (40). Additionally, the extended conformation of fucoidan provides effective steric stabilization, further enhancing colloidal stability (65). Notably, over the entire pH examined, no precipitation or sedimentation was observed in the appearance of Que-ZE-SC-FD; furthermore, the Que-ZE-SC-FD remained relatively low particle size (diameter < 180 nm) and high surface charge (absolute value of ζ-potential >20 mV), showing their colloidal stability against particle aggregation (Figure 3D). It is interesting that at pH 2, compared with Que-ZE-FD nanoparticles, Que-ZE-SC-FD exhibits better stability (smaller particle size and higher absolute potential value), indicating that SC effectively enhances the stability of Que-ZE-FD nanoparticles. Similarly, Que-ZE-SC-FD exhibited improved stability compared to Que-ZE-SC within pH 3.0–4.0, suggesting that FD contributes to stabilization in this pH range. Overall, the Que-ZE-SC-FD nanoparticles demonstrate excellent stability throughout pH 2.0–8.0, confirming the synergistic stabilizing effects of SC and FD on quercetin-loaded zein nanoparticles.

#### 3.2.2 Ionic strength stability

**Figure 4:**
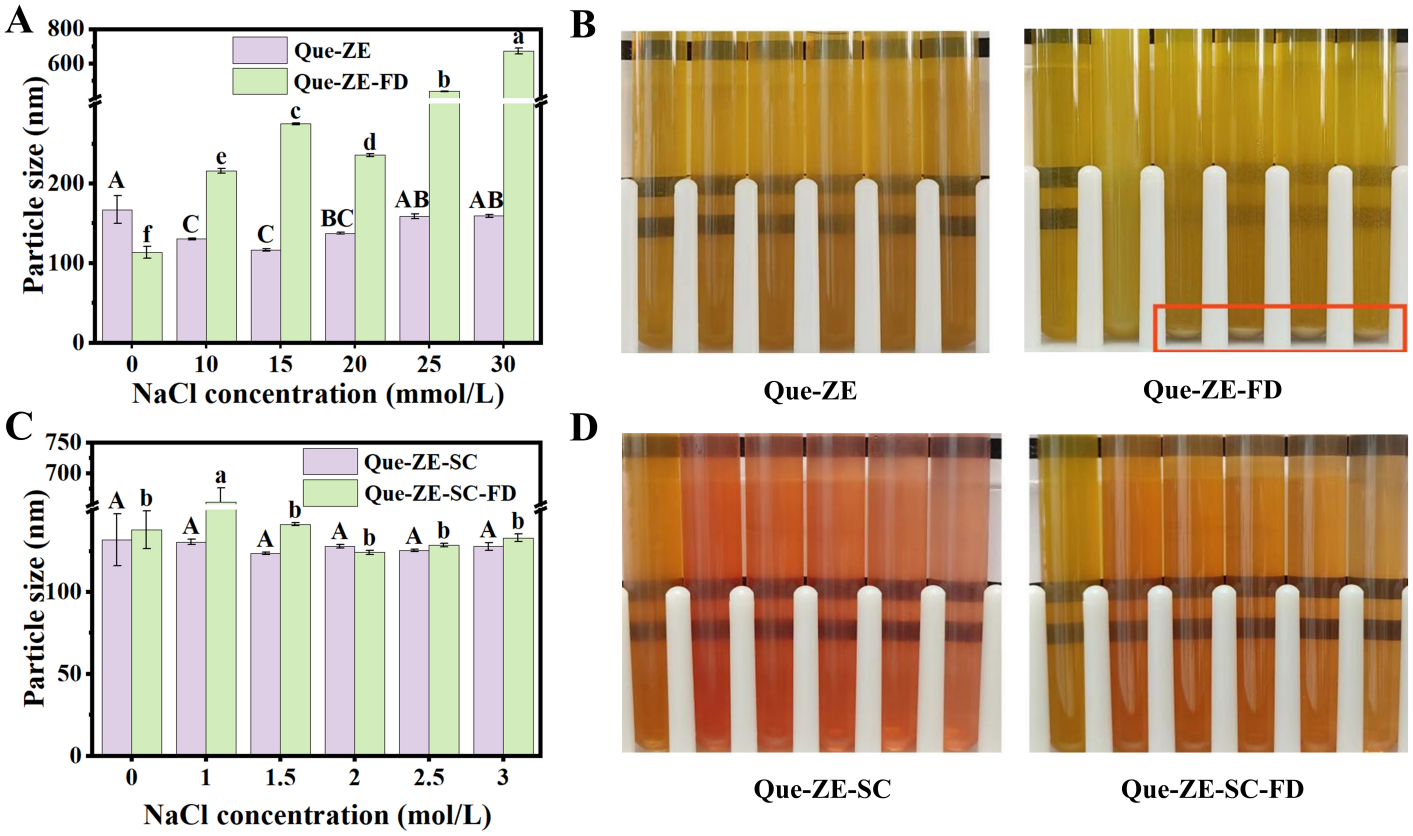
Effects of NaCl concentration on the mean particle size of Que-ZE and Que-ZE-FD nanoparticles **(A)** and their visual appearance **(B)**. Effects of NaCl concentration on the mean particle size of Que-ZE-SC and Que-ZE-SC-FD nanoparticles **(C)** and their visual appearance **(D)**. Different letters above the error bars indicate statistically significant differences (*p* < 0.05).

The particle size and appearance (Figure 4) of Que-ZE, Que-ZE-SC, Que-ZE-FD, and Que-ZE-SC-FD nanoparticles were assessed across varying NaCl concentrations. Under these ionic strengths (0–30 mmol/L), the average particle size of Que-ZE nanoparticles remained relatively stable, with no observable aggregation or precipitation. In contrast, the average particle size of Que-ZE-FD nanoparticles increased significantly (Figures 4A, B). Notably, pronounced aggregation occurred in the Que-ZE-FD nanoparticle dispersion at NaCl concentrations between 15 and 30 mmol/L. This phenomenon may be arises from the electrostatic shielding effect induced by sodium ions (66), which neutralizes the surface anionic charges and thereby reduces inter-particle electrostatic repulsion—a critical factor governing nanoparticle stability. These findings are consistent with those reported by Yuan et al. (67).

However, the addition of SC conferred excellent stability to both Que-ZE-SC and Que-ZE-SC-FD nanoparticles, even at high NaCl concentrations (1.5–3.0 mol/L; Figures 4C, D). Specifically, at 3.0 mol/L NaCl, particle size were 129.7 ± 2.7 and 127.8 ± 4.5 nm for Que-ZE-SC and Que-ZE-SC-FD nanoparticles, respectively, with no aggregation or precipitation. These results indicate that SC-stabilized zein-based nanoparticles exhibit robust stability under high ionic strength conditions. This enhanced stability may result from both electrostatic interactions and steric repulsion within the complexes (31), consistent with previous research by Liu et al. (40).

#### 3.2.3 Thermal stability

**Figure 5:**
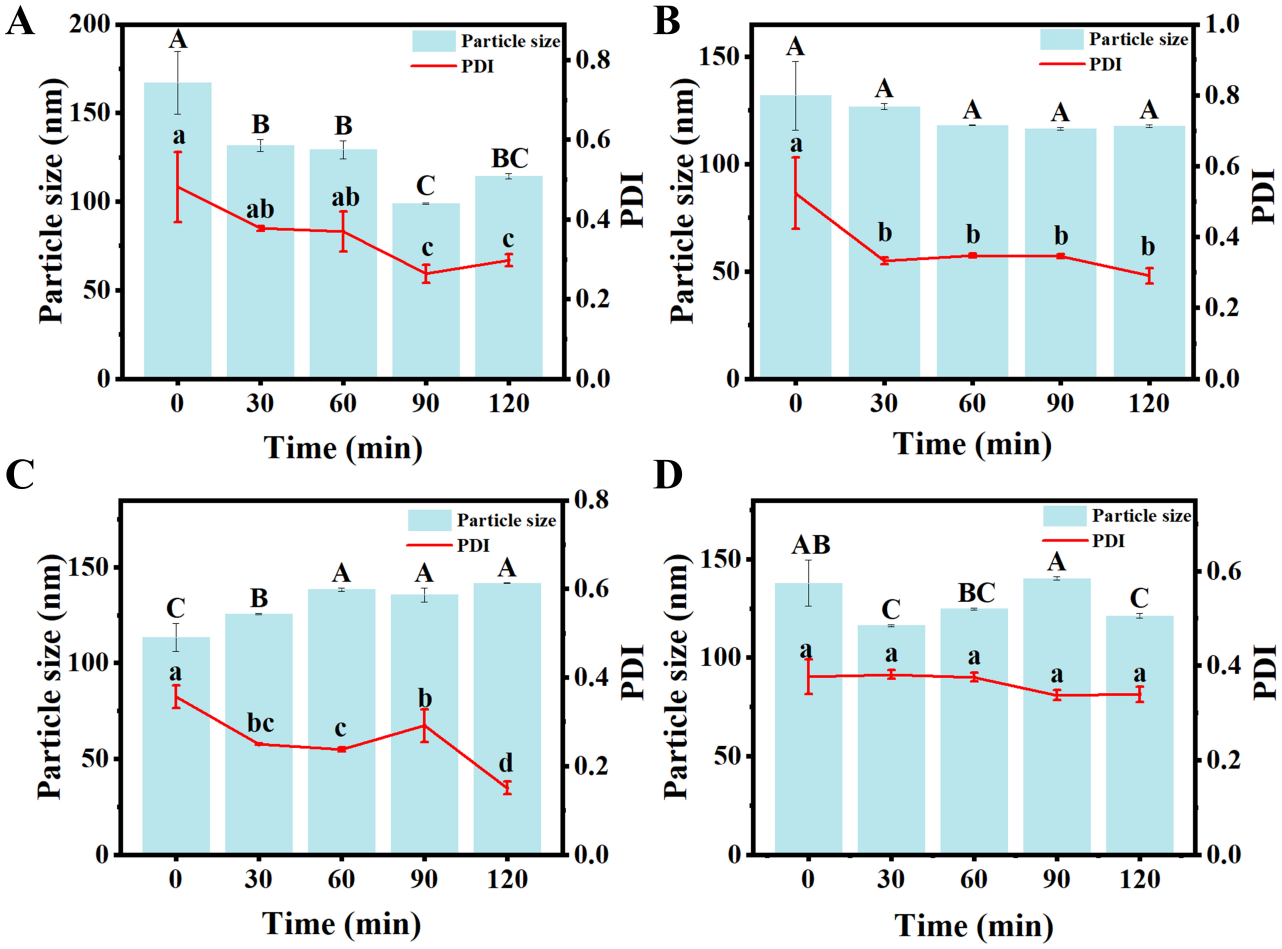
Effects of heating at 80°C (0–120 min) on the particle size and polydispersity index (PDI) of Que-ZE **(A)**, Que-ZE-SC **(B)**, Que-ZE-FD **(C)**, and Que-ZE-SC-FD **(D)** nanoparticles.

High temperature significantly affects the stability of composite nanoparticles, which is a critical factor for food storage applications. As shown in Figure 5, after heating at 80°C for 120 min, the particle sizes of four nanoparticle types—Que-ZE, Que-ZE-SC, Que-ZE-FD, and Que-ZE-SC-FD—remained stable, measuring 130.5 ± 12.3, 118.6 ± 17.4, 147.8 ± 4.6, and 129.1 ± 12.2 nm, respectively. These results are consistent with the findings reported by Ma et al. (68). Notably, the particle size of Que-ZE-SC-FD nanoparticles decreased slightly from 137.8 ± 11.6 to 129.1± 12.2 nm, while the PDI decreased from 0.37 ± 0.04 to 0.22 ± 0.02. These changes, which align with (45), suggest improved system homogeneity. The observed thermal stability may be attributed to zein's denaturation temperature, which occurs near 100°C (67). Overall, these findings demonstrate that Que-ZE-SC-FD nanoparticles possess excellent thermal stability under high-temperature conditions.

#### 3.2.4 Long-term storage stability

**Figure 6:**
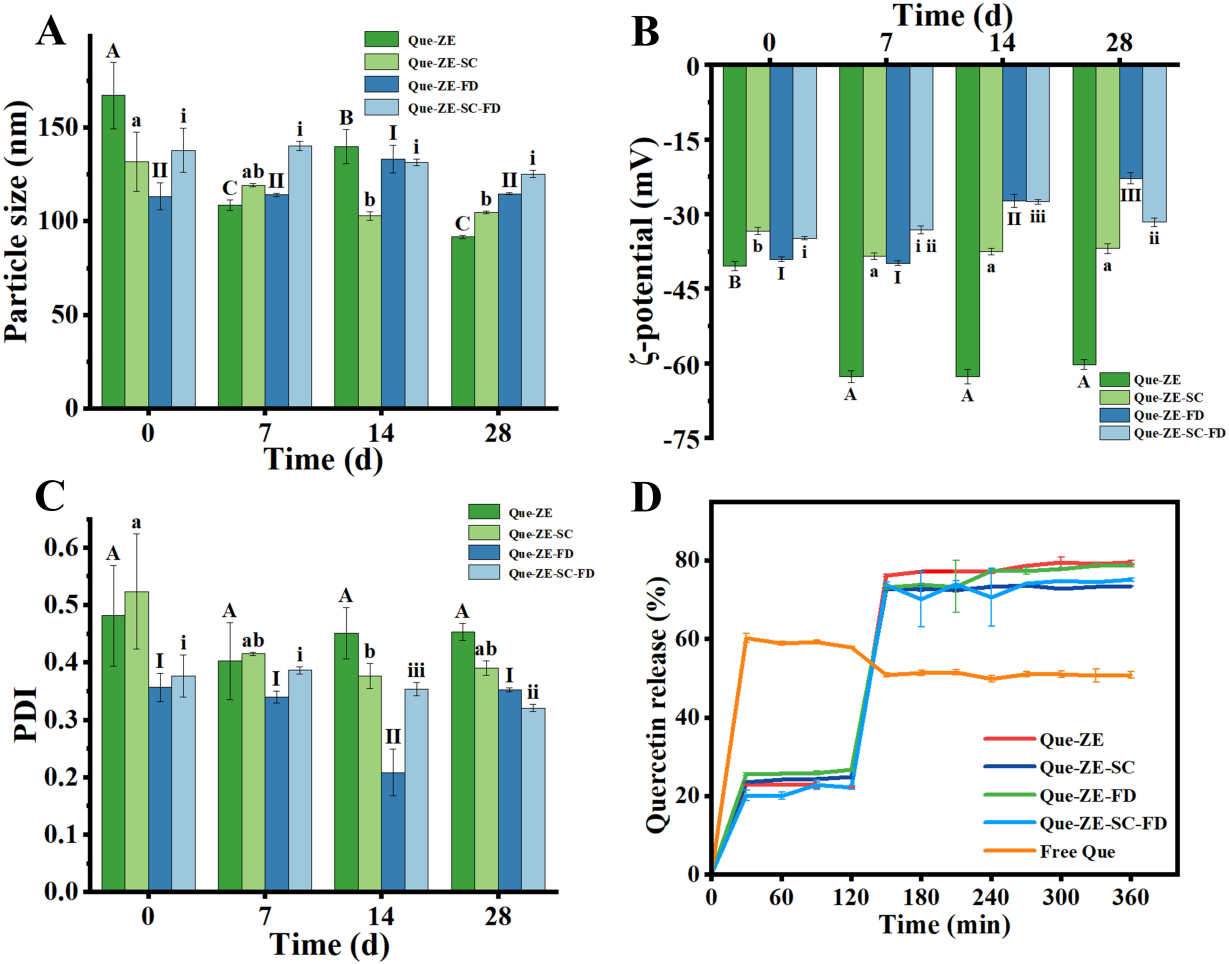
The effects of 28-day storage on the particle size **(A)**, ζ-potential **(B)**, and PDI **(C)** of four types of nanoparticles (Que-ZE, Que-ZE-SC, Que-ZE-FD, and Que-ZE-SC-FD); release profiles of quercetin from Que-ZE, Que-ZE-SC, Que-ZE-FD, and Que-ZE-SC-FD nanoparticles during simulated gastrointestinal digestion **(D)**.

The significance of storage stability in delivery systems lies in how it directly affects the longevity of the bioactive substances. The surface charge characteristics of nanoparticles can be altered due to the chemical degradation or movement of charged components The particle size, ζ-potential, and PDI of four nanoparticle formulations (Que-ZE, Que-ZE-SC, Que-ZE-FD, and Que-ZE-SC-FD) were evaluated over 28 d at 4°C in the dark (Figures 6A–C) to assess their potential shelf life in commercial products. The Free Que suspension was highly unstable, exhibiting extensive aggregation and precipitation during storage. As shown in Figure 6A, Que-ZE nanoparticles exhibited a significant particle size reduction from 167.0 ± 17.6 nm to 91.6 ± 0.9 nm during storage, suggesting potential structural degradation or disintegration. The lower storage stability of ZE-Que nanoparticles could be caused by hydrophobic aggregation between nanoparticles (14). Que-ZE-SC-FD nanoparticles demonstrated remarkable stability, with particle size reducing slightly from 137.8 ± 11.6 nm to 125.0 ± 1.9 nm over the same period. Correspondingly, Que-ZE-SC-FD maintained a lower PDI (0.32 ± 0.01 at day 28) compared to the other formulations. These results indicate that the combined use of sodium caseinate and fucoidan enhances nanoparticle stability, likely through synergistic electrostatic interactions and steric repulsion within the complexes. Changes in ζ-Potential are presented in Figure 6B. The absolute ζ-potential values of Que-ZE-SC, Que-ZE-FD, and Que-ZE-SC-FD decreased during storage, potentially due to a reduction in surface charge that diminished inter-particle electrostatic repulsion (69). This trend is consistent with the observations by Chen et al. (70), who reported a similar decline in ζ-potential for Cur-Zein-Que-HA nanoparticles after 6 months of storage, attributed to changes in interfacial composition or nanoparticle structure. Notably, Que-ZE displayed a transient increase in ζ-potential on day 7, possibly resulting from precipitate formation that affected surface charge measurement. Furthermore, sodium caseinate and fucoidan increase the hydrophilicity of zein-based nanoparticles, thereby reducing aggregation-prone hydrophobic interactions. By forming an outer protective layer, these biomolecules also serve as a physical barrier against light and oxygen, thus helping prevent quercetin degradation (65, 71).

### 3.3 *In vitro* simulated gastrointestinal digestion

The digestive stability of bioactive compound delivery systems is critical for the effective intestinal delivery. We therefore evaluated four Que-loaded nanoparticles (Que-ZE, Que-ZE-SC, Que-ZE-FD, and Que-ZE-SC-FD) and free quercetin using an *in vitro* gastrointestinal model. As shown in Figure 6D, free quercetin exhibited rapid release (57.9 ± 0.2%) during the simulated gastric fluid (SGF) phase (0–120 min). In contrast, nanoparticle formulations showed significantly lower release rates at 120 min: Que-ZE (22.1 ± 0.2%), Que-ZE-SC (24.8 ± 0.3%), Que-ZE-FD (26.6 ± 0.3%), and Que-ZE-SC-FD (22.2 ± 0.5%). The release mainly originates from the particle surface. The minimal release from Que-ZE-SC-FD nanoparticles likely reflects synergistic protection by sodium caseinate and fucoidan. Under acidic SGF conditions, nanoparticle aggregation limited pepsin access to inner layers, further restricting quercetin release. Upon transition to the simulated intestinal fluid (SIF), all nanoparticles exhibited accelerated quercetin release—consistent with Liu et al. (72)—attributed to bile salts, fatty acids, and peptides disrupting nanoparticle structure. Conversely, free quercetin release slowed (final 50.8 ± 0.9%), aligning with studies on quercetin re-crystallization in intestinal media (73). pH fluctuations during digestion may also promote quercetin oxidation and degradation (63), compounded by intestinal metabolism into phenolic metabolites (74). Final quercetin release rates were Que-ZE (79.4 ± 0.6%), Que-ZE-SC (73.5 ± 0.2 %), Que-ZE-FD (78.7 ± 0.4%), and Que-ZE-SC-FD (75.0 ± 0.5%). This sustained release profile—contrasting sharply with free quercetin—enhances quercetin transport and absorption, thereby improving its overall bioavailability (69, 75). In our study, the encapsulated quercetin exhibited higher release for several reasons. Firstly, it was recognized that hydrophobic substances in their amorphous state exhibit a greater dissolution rate, solubility, release and bioaccessibility compared to their crystalline counterparts (76). Secondly, the breakdown of zein nanoparticles might result in the formation of peptides that are soluble in water, which could attach to and potentially dissolve the quercetin. Lastly, the increased surface area of the nanoparticles could have resulted in a quicker release of quercetin into the adjacent gastrointestinal fluids (77). Overall, our study highlighted the potential of quercetin as a preferable delivery system to improve the bio-accessibility of quercetin.

## 4 Conclusion

In this study, a ZE-SC-FD nanoparticle delivery system loaded with quercetin was successfully developed using a simple, green, and safe pH-driven self-assembly method. Hydrogen bonding, electrostatic interactions, and hydrophobic interactions occur among ZE, SC, FD, and Que. Furthermore, hydrogen bonds, steric effects, electrostatic interactions (attraction among zein, sodium caseinate, and fucoidan; repulsion between nanoparticles), and hydrophobic interactions played a driving role in the formation of the Que-ZE-SC-FD nanoparticles. Among the four nanoparticles (Que-ZE, Que-ZE-SC, Que-ZE-FD, and Que-ZE-SC-FD), Que-ZE-SC-FD demonstrates excellent encapsulation efficiency, high pH and ionic stability, as well as thermal stability, enabling long-term storage. *In vitro* simulated digestion experiments revealed that Que-ZE-SC-FD nanoparticles can effectively regulate the delivery and release of quercetin, thereby improving its bioavailability. These findings provide valuable insights for designing food-grade colloidal carriers to encapsulate, protect, and deliver quercetin or other bioactive compounds. However, the *in vivo* bioavailability and absorption mechanisms of quercetin delivered via ZE-SC-FD nanoparticles require comprehensive assessment in future studies. However, this study does not include *in vivo* validation to substantiate the proposed enhancement in bioavailability.

## Data Availability

The raw data supporting the conclusions of this article will be made available by the authors, without undue reservation.
